# PASCOM

**DOI:** 10.1186/1757-1146-4-S1-I8

**Published:** 2011-05-20

**Authors:** Trevor D Prior

**Affiliations:** 1 Homerton University Hospital, London, E9 6SR, UK

## 

PASCOM (Podiatric Audit in Surgery and Clinical Outcome Measure) is the national audit system used by the fellows of the Faculty of Podiatric Surgery, College of Podiatry, Society of Chiropodists and Podiatrists. This was initially developed as a 3 part audit system which captured data relating to the surgical event, post operative complications / outcome and patient satisfaction. Whilst this provided a wealth of data, there were limitations. In particular, data was captured locally and relied on centres submitting information. Furthermore, for training centres, it was more difficult to identify the role / interaction of the tutor and trainee. Extensive update with funding from the Society of Chiropodists and Podiatrists has enabled the development of Pascom-10. This is now a web based system which not only captures the previous data but also facilitates capture of outpatient information, injection therapy and includes a patient related outcome measure. Furthermore, it has been expanded to allow the general membership of the profession to record data relating to injection therapy and nail / skin surgery. This presentation will outline the original system and subsequent developments and detail how it can be integrated into practice.

